# *Porphyromonas gingivalis* in Colorectal Cancer and its Association to Patient Prognosis

**DOI:** 10.7150/jca.83395

**Published:** 2023-05-21

**Authors:** Maïwenn Kerdreux, Sofia Edin, Thyra Löwenmark, Vicky Bronnec, Anna Löfgren-Burström, Carl Zingmark, Ingrid Ljuslinder, Richard Palmqvist, Agnes Ling

**Affiliations:** 1 Department of Medical Biosciences, Pathology, Umeå University, Umeå, Sweden.; 2 Department of Radiation Sciences, Oncology, Umeå University, Umeå, Sweden.

**Keywords:** *Porphyromonas gingivalis*, colorectal cancer, microbiota, survival

## Abstract

Microbiota dysbiosis may affect both the development and progression of colorectal cancer (CRC). Large metagenomic studies have highlighted specific oral bacteria linked to CRC including *Porphyromonas gingivalis*. Few studies have however analysed the implications of this bacterium in CRC progression and survival. In this study, we investigated the intestinal presence of *P. gingivalis* by qPCR in both faecal and mucosal samples from two different patient cohorts, including patients with precancerous dysplasia or CRC, as well as controls. *P. gingivalis* was detected in 2.6-5.3% of CRC patients and significantly different levels of *P. gingivalis* were found in faeces of CRC patients compared to controls (*P* = 0.028). Furthermore, an association was found between the presence of *P. gingivalis* in faeces and tumour tissue (*P* < 0.001). Our findings further suggested a potential link between mucosal *P. gingivalis* and tumours of MSI subtype (*P* = 0.040). Last but not least, patients with faecal *P. gingivalis* were found to have a significantly decreased cancer-specific survival (*P* = 0.040). In conclusion, P. gingivalis could be linked to patients with CRC and to a worse patient prognosis. Further studies are needed to elucidate the role of P. gingivalis in CRC pathogenesis.

## Introduction

Colorectal cancer (CRC) is the third most common cancer worldwide and the second most common cause of cancer death 1. It is a heterogenous disease, but with a relatively low level of hereditability, which reflects the importance of the environment in development of sporadic CRC.

The colon is highly colonised by microorganisms and harbours around 70% of the human microbiome 2. The gut microbiota is known to play an important role in mucosal homeostasis, nutritional absorption, immunity, epithelial barrier function as well as carcinogenesis 3. Recent metagenomic studies have established specific alterations in the gut microbiota associated with CRC 4. Meta-analyses have further identified a microbial core of enriched bacteria in CRC including bacteria from the oral taxa such as *Fusobacterium nucleatum* and *Parvimonas micra*, as well as bacteria from the *Porphyromonas* genus 5, 6. A driver/passenger theory has been suggested in CRC progression, where some gut commensal bacteria may be responsible for the overexpression of pro-inflammatory cytokines and the induction of chronic inflammation, which may trigger the development of adenoma followed by adenocarcinoma 7, 8. Driver bacteria may also directly trigger cancer development by inducing epithelial DNA damage. Succeeding tumourigenesis, the environment is transformed, leading to an overgrowth of opportunistic bacteria. These passenger bacteria may replace the driver bacteria - as they benefit from a growth advantage in the tumour microenvironment - and cause inflammation at a later stage contributing to cancer progression. A good illustration of a potential driver is *Escherichia coli,* where some strains are capable of releasing the toxin colibactin. This toxin can cause double stranded DNA breaks of epithelial cells which subsequently may lead to tumourigenesis 9. The pathogenic colibactin-expressing *E. coli* has been shown to be increased in CRC 10. Other potential driver/passengers include *F. nucleatum* and* P. micra* from the oral microbiota*. F. nucleatum* has been found interacting with different aspects of CRC progression, in both preclinical and clinical studies (reviewed in 11), and has been associated with decreased patient survival 12, and resistance to chemotherapy 13. *P. micra* is also an interesting bacterium, shown to be associated with tumour immune profiles in CRC and to negatively impact patient survival 14-16.

There are at least three different evolutionary routes described in sporadic colorectal cancer 17. The traditional pathway occurs in 50% to 70% of the cases and results in the transition from a normal mucosa to an adenoma and further to an adenocarcinoma. This pathway is caused by mutations in tumour oncogenes and suppressor genes (*e.g. APC*, *KRAS*, and *TP53*) and results in chromosomal instability 17. In the serrated pathway, which occurs in 10% to 20% of the cases, the normal mucosa evolves into sessile serrated lesions (SSL) often with mutations in the *BRAF* proto-oncogene, followed by the development into an adenocarcinoma through MutL homolog 1 (MLH1) promoter methylation leading to microsatellite instability (MSI). While the traditional pathway occurs more often in the distal colon, the serrated pathway is found mostly in the proximal colon and linked to a good prognosis. Understanding the interactions of the microbiota with these developmental pathways is currently an important objective in colorectal cancer research.

In this study, the aim was to investigate another oral pathogen, *P. gingivalis*, and its associations with CRC progression and patient survival. *P. gingivalis* was selected as a putative driver/passenger bacteria in CRC since it has previously been linked to periodontitis, an inflammatory condition in the oral cavity 18, as well as to clinical stages and survival in oral and esophageal cancers 19. Analyses were conducted to identify *P. gingivalis* in faecal samples from patients with precancerous dysplasia or CRC using two different patient cohorts. For some of the patients, the presence of *P. gingivalis* was also analysed in tumour tissue.

## Materials and methods

### Study patients

The study was based on patients from the Uppsala-Umeå Comprehensive Cancer Consortium (U-CAN) cohort of CRC patients 20, and the Fecal and Endoscopic Colorectal Study in Umeå (FECSU) 21. The collection of patients for the U-CAN cohort was initiated in 2010 and includes patients diagnosed with CRC with longitudinally collected blood, tissue, faeces, radiological and clinical data. The FECSU cohort consists of patients who underwent colonoscopy at Umeå University hospital between the years 2008-2013, due to gastrointestinal symptoms and includes patients with dysplasia or CRC, but also patients without pathological findings.

For this study, 257 CRC patients (stage I-IV) diagnosed between the years 2010-2014 were included from U-CAN, as well as 135 patients with dysplasia and 39 patients with CRC from the FECSU cohort. Controls were selected from the FECSU cohort and were matched by age and gender from the patients with no neoplastic findings during colonoscopy, as previously described 22. Inclusion and exclusion criteria, as well as sample collection procedures, for the U-CAN 22 and FECSU 21 cohorts were previously described. For all patients included in this study, faecal samples were collected at time of diagnosis and before start of treatment. Fresh frozen tumour tissue specimens were also collected for CRC patients from the U-CAN cohort (n=115).

The study protocol was approved by the Regional Ethical Review Board in Umeå, Sweden (dnr 2016/219-31 and dnr 08-184M), including the procedure by which the patients gave informed consent.

### Detection of *P. gingivalis* by Quantitative real time PCR

The QIAamp PowerFecal DNA kit (Qiagen, Sollentuna, Sweden) was used to extract gDNA from approximately 0.2 g of stool, according to manufacturer's instructions. Fresh frozen tumour tissues were homogenised using a Precellys 24 homogenizer (Bertin Techologies, Rockville, MD, USA) and gDNA was extracted using the Allprep DNA/RNA/miRNA Universal kit (Qiagen). Double stranded DNA-recovery was measured using the Qubit^®^ dsDNA BR Assay (Invitrogen, Carlsbad, CA, USA) with the Qubit^®^ 2.0 Fluorometer (Invitrogen). Quantitative real time PCR (qPCR) was used to detect *P. gingivalis* in DNA extracted from faecal samples or fresh frozen tumour tissues using the Quant-Studio^TM^ 6 Flex Real-Time PCR system (Applied Biosystems, Foster City, CA, USA). *P. gingivalis* was analysed according to previous studies 23, 24, to quantify the gene encoding the small subunit of 16S ribosomal RNA of *P. gingivalis*. Genomic DNA from *P. gingivalis* strain 2561 ATCC 33277 (LGC Standards AB, Bora, Sweden) was used as a positive control. The following primers and probes were used: 5′-GCGCTCAACGTTCAGCC-3′ (forward); 5′-CACGAATTCCGCCTGC-3' (reverse) and FAM-CACTGAACTCAAGCCCGGCAGTTTCAA-TAMRA (probe). Reactions were run using the TaqMan Universal PCR Mix (Applied Biosystems, Warrington, UK) with 10 µM of primers and 10 ng of gDNA template in a total volume of 20 µL. The cycle conditions used were: 2 minutes at 50°C, 10 minutes at 95°C, followed by 40 cycles of: 95°C for 15 seconds, 60°C for 60 seconds. Data was collected from stable duplicates (standard deviation < 0.5). As an internal criterion, a positive *P. gingivalis* status was given to samples amplified within 38 cycles. The levels of *P. gingivalis* were presented as a relative quantification using the 2^-ΔCq^ method. 16SrRNA and the human gene *PGT* were used as reference for faeces and fresh frozen tissue, respectively, as previously described 22. Exclusions included depleted samples, low DNA yield, or poor sample quality. After exclusions, analyses were performed on faecal samples from 247 CRC patients from the U-CAN cohort and 89 matched controls (from FECSU), as well as on faecal samples from 128 patients with dysplasia, 38 patients with CRC, and 61 controls from the FECSU cohort. Fresh frozen tumour tissues were analysed from 113 CRC patients. The analyses of *P. micra* and *F. nucleatum* in the U-CAN patients were previously described 22.

### Statistical methods

IBM SPSS Statistics 28 was used for statistical analyses (SPSS Inc.). The Fischer´s exact test was used for comparisons between categorical variables and the Mann-Whitney U test or Kruskall-Wallis test were used to compare differences in continuous variables between groups. Kaplan-Meier survival analysis was used to estimate cancer-specific survival, with the log-rank test used to compare differences in outcome between groups. Patients were followed from the time of surgery to the time of death or end of follow-up (October 2021). Cancer-specific death was defined as death with known disseminated or recurrent disease. Patients not surgically resected for CRC and patients dying from post-operative complications within 90 days were excluded from survival analyses. Multivariable survival analysis was performed using Cox proportional hazard models. *P* < 0.05 was used to consider statistical significance.

## Results

### Significantly different levels of *P. gingivalis* in faecal samples from CRC patients compared to controls

**Figure 1:**
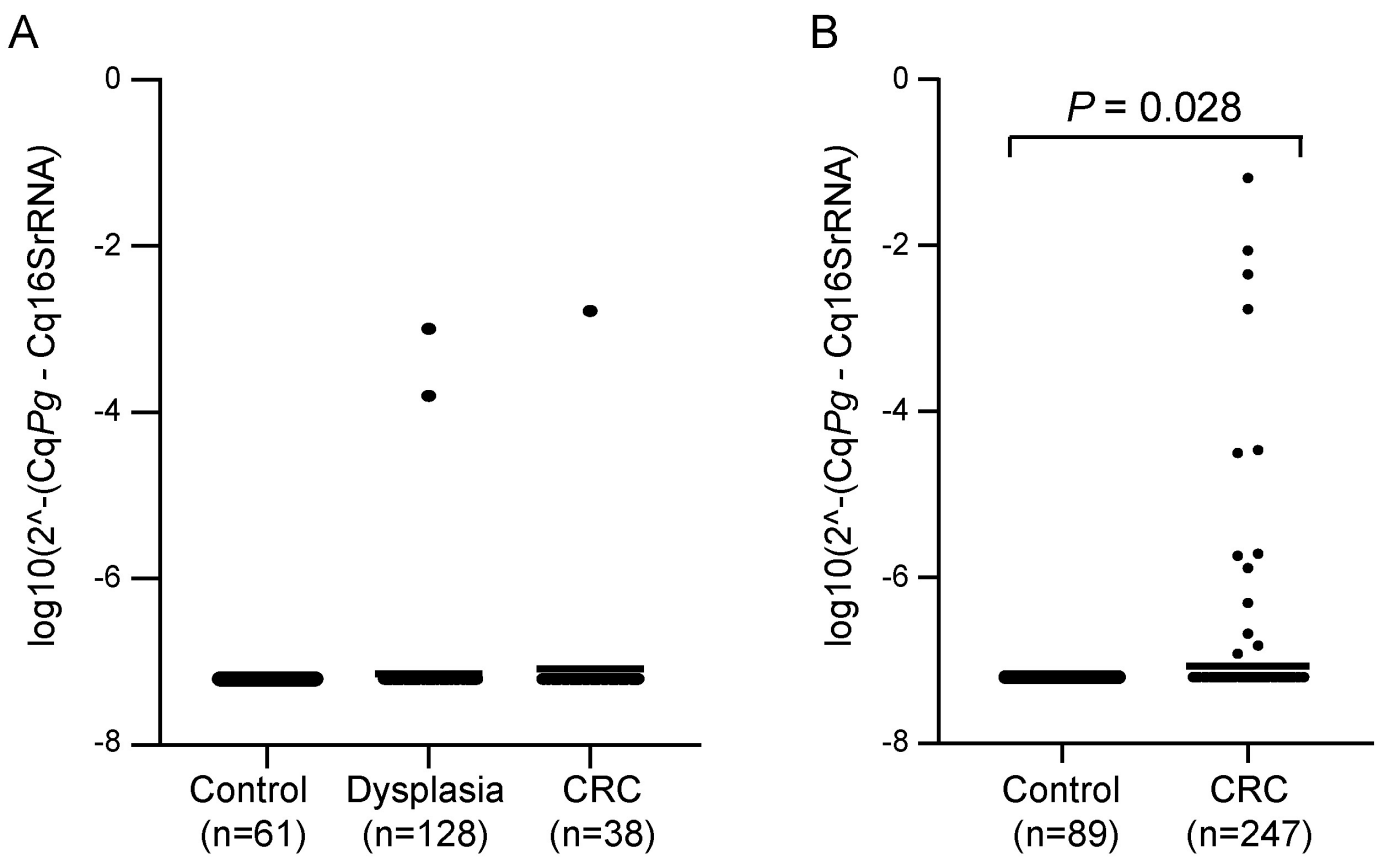
** Levels of *P. gingivalis* in faecal samples from patients with dysplasia or CRC *versus* controls.** Scatter plots showing the relative levels of *P. gingivalis* (*Pg*) analysed by qPCR in faecal samples from patients with dysplasia or CRC and controls in (A) the FECSU cohort and (B) the U-CAN cohort. Horizontal lines indicate mean relative expression. A Mann-Whitney test was used for the statistical comparison of *P. gingivalis* levels between CRC patients and controls.

Faecal samples from CRC patients and control individuals (without neoplastic findings) were analysed for the presence of *P. gingivalis* by qPCR in two different patient cohorts. For the FECSU cohort, two out of 128 patients (1.6%) with dysplasia and one out of 38 patients (2.6%) with CRC were positive for *P. gingivalis* (Figure 1A). Among the 247 CRC patients from the U-CAN cohort, 13 faecal samples (5.3%) were positive for *P. gingivalis*, and the levels of *P. gingivalis* were found to be significantly different in faecal samples from CRC patients compared to controls (*P* = 0.028) (Figure 1B). No *P. gingivalis* positive samples were found among the controls. Since so few cases were found positive in the FECSU cohort, we continued our studies focusing on the patients included from the U-CAN cohort.

To assess the presence of *P. gingivalis* in the colorectal mucosa, we analysed fresh frozen tumour tissues from 113 U-CAN CRC patients. In total, seven samples (6.2%) were positive for mucosal *P. gingivalis,* and five out of these samples were also positive for the bacterium in faeces. Furthermore, a significant association in distribution of *P. gingivalis* between these two compartments was found (Table 1).

### Associations between *P. gingivalis* and clinicopathological and tumour molecular characteristics

We investigated the associations of *P. gingivalis* in faeces or tumour tissue of U-CAN CRC patients (n=247) to clinical and pathological characteristics (Table 2). We found a slight association of faecal *P. gingivalis* to patient age, but no significant associations were found with gender or tumour location, stage, grade, or type (mucinous/non-mucinous), neither for faecal nor for mucosal *P. gingivalis*. Furthermore, *P. gingivalis* was not significantly associated with perineural or venous invasion. Nevertheless, the presence of *P. gingivalis* in tumour tissue was associated with a mucinous tumour type (*P* = 0.045).

We next analysed the presence of *P. gingivalis* in relation to tumour molecular characteristics. We could not find any significant associations to molecular characteristics for faecal *P. gingivalis*. However, even though limited by sample size, we did find a significant association between the presence of *P. gingivalis* in tumour tissue and tumours of MSI subtype (*P* = 0.040) (Table 3).

### Associations between *P. gingivalis* and other oral CRC-associated bacteria

We further explored the interactions of *P. gingivalis* with *F. nucleatum* and *P. micra*. We found no association of *P. gingivalis* with these bacteria in faecal samples. However, we observed a significant association between the presence of *P. gingivalis* and *P. micra* in tumour tissue (*P* = 0.005) (Table 4).

### Faecal detection of *P. gingivalis* is associated with decreased patient survival

**Figure 2:**
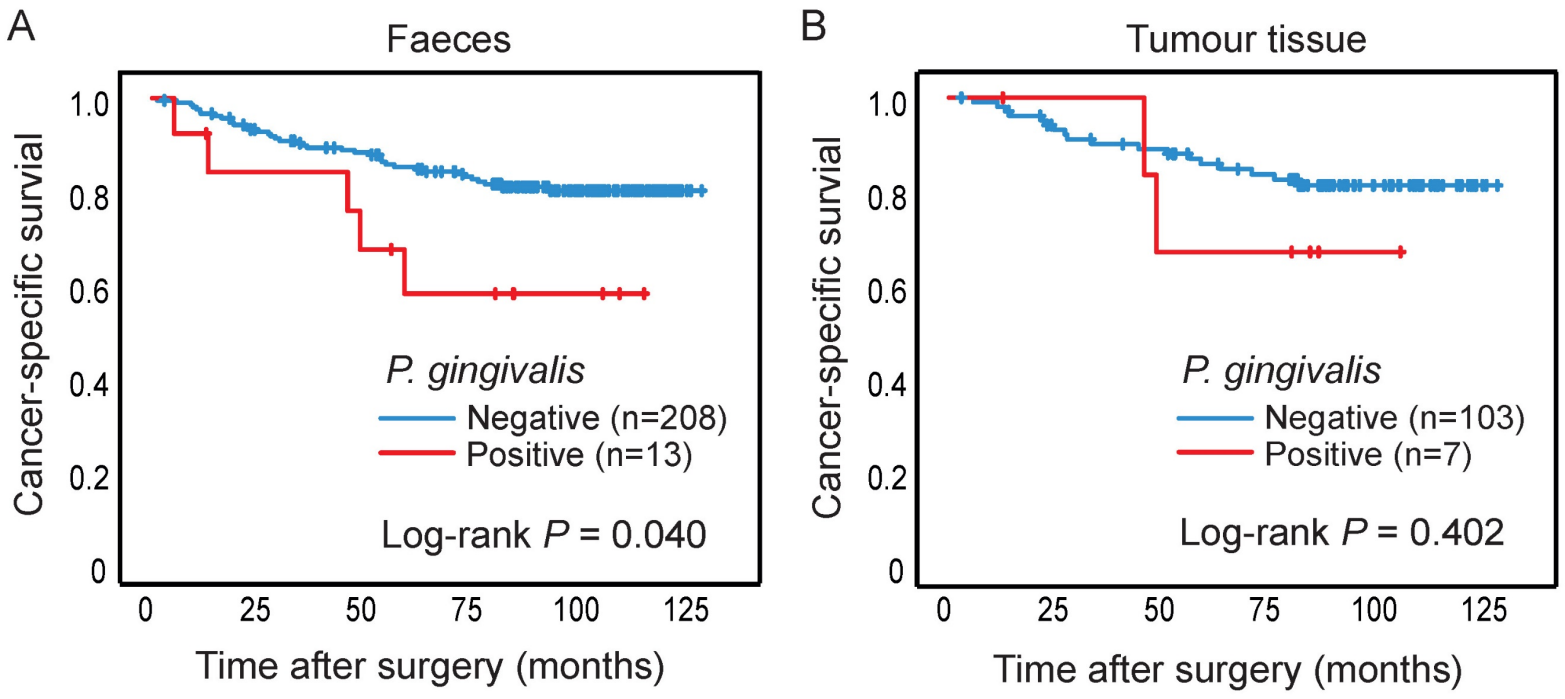
** The presence of *P. gingivalis* in relation to survival of CRC patients.** Kaplan-Meier plots of cancer-specific survival in patients negative or positive for *P. gingivalis* in (A) faeces or (B) tumour tissue. Log-rank tests were used to calculate *P* values*.*

The presence of *P. gingivalis* in faeces and tumour tissue was analysed in relation to cancer-specific patient survival, and patients with *P. gingivalis* positive faecal samples were found to have a significantly worse prognosis compared to patients with *P. gingivalis* negative samples (*P* = 0.040) (Figure 2A). The significance of the prognostic role of faecal *P. gingivalis* was maintained in a multivariable Cox proportional hazard model including age, gender, and tumor stage (hazard ratio (HR) = 2.90, CI 1.01-8.32, *P* = 0.047)). No significant difference in survival was found according to *P. gingivalis* in tumour tissue (Figure 2B).

## Discussion

Recent research suggests the involvement of oral microbes in the development and progression of CRC 25. In this study, we explored the role of the oral bacteria *P. gingivalis* in CRC, using two different patient cohorts with collected faecal and tumour tissue samples.

We found a significant difference of *P. gingivalis* faecal levels between CRC patients and non-cancerous controls. *P. gingivalis* was not detected in the controls, but in some samples from patients with pre-cancerous lesions, suggesting that intestinal presence of *P. gingivalis* could in some cases be an early event. We further found an association between the presence of faecal and mucosal *P. gingivalis*. Our findings are in line with previous studies on CRC using both 16SrRNA sequencing and qPCR to identify *P. gingivalis* 26, 27. We found a weak association of mucosal *P. gingivalis* with tumours of MSI subtype. We further found slight associations of *P. gingivalis* to other oral CRC-associated bacteria in tumour tissue, in particular *P. micra*. *P. gingivalis* has previously been related to tumours of CMS1 subtype (including tumours of MSI subtype) together with *F. nucleatum* and *P. micra* 28*,* which is in line with our finding of a putative association between *P. gingivalis* with these bacteria in CRC. Further larger studies on the relation of *P. gingivalis* to CRC molecular traits are needed to establish these associations. Interestingly, *P. gingivalis* has been shown to synergistically promote extra-gastrointestinal infection and to form biofilm together with other oral bacteria in periodontitis 18, 29. Oral microbiota has also been linked to biofilm formation in CRC 30, 31. In fact, CRC-associated *F. nucleatum* has been shown to potentially originate from the oral cavity 32. Therefore, collecting clinical data about the oral health of the patients would be an important topic for future CRC studies.

The impact of *P. gingivalis* on cancer-specific patient survival was next investigated. Patients with faecal *P. gingivalis* - even though few - were found to have a significantly decreased survival compared to patients without faecal *P. gingivalis*. Our findings were corroborated in a study by Wang *et al.,* where they found that *P. gingivalis* was enriched in tumour tissue and faecal samples from CRC patients and that its presence was associated with a poor prognosis 27. Moreover, *P. gingivalis* has previously been linked to lymph node metastasis and survival in oral and oesophageal cancer 19. Survival in CRC has also been shown to be impacted by the presence of *F. nucleatum* and* P. micra* 12, 14, 16, further supporting a possible interaction between these bacteria in CRC.

Although this study included a relatively large cohort of patients with stool samples, few samples were found positive for *P. gingivalis*. Studies using larger cohorts to increase the power of statistical tests are required, especially when studying the relation to subgroups of CRC. In this study, we found that when *P. gingivalis* was identified in faecal samples, it was often present also in tumour tissue, but the precise location within the tumours remains to be explored. Further studies are also needed to address the cause-and-effect relationship of this bacteria in CRC pathogenesis, including mouse models and/or *in vitro* experiments to explore the molecular mechanisms behind. Few possible mechanisms for *P. gingivalis* in CRC progression have been described. Using *in vitro* experiments and mouse models, Okumura *et al.*, reported an increased production of butyrate - a short chain fatty acid reported to enhance tumourigenesis - by *P. gingivalis* leading to cellular senescence and the onset of CRC tumours 26. Wang *et al.,* further found that *P. gingivalis* could promote CRC by NLRP3 inflammasome activation both *in vitro* and *in vivo* 27.

In summary, we found that *P. gingivalis* was associated with CRC and with a worse patient prognosis. A possible interaction between *P. gingivalis* with other oral CRC-associated bacteria and tumours of MSI subtype was further implied, but these putative interactions need to be further explored using larger patient cohorts. An increased understanding of the pathogenesis of CRC may lead to the identification of potential screening markers, as well as important improvements in personalised medicine.

## Figures and Tables

**Table 1:** Cross-tabulation between levels of *P. gingivalis* in faeces and tumour tissue of CRC patients.

	*P. gingivalis* in tumour tissue		
	Negative (n=106)	Positive (n=7)	*P* value
***P. gingivalis* in faeces, n (%)**			< 0.001
Negative (n=234)	102 (98.1)	2 (1.9)	
Positive (n=13)	4 (44.4)	5 (55.6)	

Fisher's exact test was used for comparisons of categorical variables.

**Table 2:** Clinicopathological characteristics of study patients in relation to *P. gingivalis* in faeces and tumour tissue of CRC patients.

	*P. gingivalis* in faeces			*P. gingivalis* in tumour tissue		
	Negative	Positive	*P* value	Negative	Positive	*P* value
**Age, n (%)**			0.038			0.778
≤ 59	40 (95.2%)	2 (4.8%)		17 (94.4%)	1 (5.6%)	
60-69	90 (97.8%)	2 (2.2%)		35 (94.6%)	2 (5.4%)	
70-79	73 (89.0%)	9 (11.0%)		38 (90.5%)	4 (9.5%)	
≥ 80	31 (100%)	0 (0%)		16 (100%)	0 (0%)	
**Gender, n (%)**			0.385			0.241
Female	92 (92.9%)	7 (7.1%)		46 (90.2%)	5 (9.8%)	
Male	142 (95.9%)	6 (4.1%)		60 (96.8%)	2 (3.2%)	
**Location, n (%)**			1.000			0.375
Right colon	49 (94.2%)	3 (5.8%)		30 (90.9%)	3 (9.1%)	
Left colon	39 (95.1%)	2 (4.9%)		19 (90.5%)	2 (9.5%)	
Rectum	146 (94.8%)	8 (5.2%)		57 (96.6%)	2 (3.4%)	
**Stage, n (%)**			0.953			0.164
I	47 (95.9%)	2 (4.1%)		32 (100%)	0 (0%)	
II	77 (93.9%)	5 (6.1%)		38 (95%)	2 (5%)	
III	65 (95.6%)	3 (4.4%)		27 (87.1%)	4 (12.9%)	
IV	36 (94.7%)	2 (5.3%)		9 (100%)	0 (0%)	
**Tumour grade, n (%)**			0.211			0.295
High grade	26 (89.7%)	3 (10.3%)		18 (90%)	2 (10%)	
Low grade	176 (95.1%)	9 (4.9%)		87 (95.6%)	4 (4.4%)	
**Tumour type, n (%)**			0.642			0.045
Non-mucinous	180 (94.7%)	10 (5.3%)		91 (96.8%)	3 (3.2%)	
Mucinous	24 (92.3%)	2 (7.7%)		14 (82.4%)	3 (17.6%)	
**Perineural invasion, n (%)**			0.083			0.272
Yes	28 (87.5%)	4 (12.5%)		17 (89.5%)	2 (10.5%)	
No	176 (95.7%)	8 (4.3%)		88 (95.7%)	4 (4.3%)	
**Venous invasion, n (%)**			0.137			0.623
Yes	41 (89.1%)	5 (10.9%)		24 (92.3%)	2 (7.7%)	
No	162 (95.9%)	7 (4.1%)		81 (95.3%)	4 (4.7%)	

Fisher´s exact test was used for comparisons of categorical variables. Missing cases were present for the following variables in faeces analyses: stage, 10 cases; tumour grade, 33 cases; tumour type and perineural invasion, 31 cases; venous invasion, 32 cases. Missing cases in tumour tissue analyses: stage, 1 case; tumour grade, tumour type, perineural invasion and venous invasion, 2 cases.

**Table 3:** Molecular characteristics of study patients in relation to *P. gingivalis* in faeces and tumour tissue of CRC patients.

	*P. gingivalis* in faeces			*P. gingivalis* in tumour tissue		
	Negative	Positive	*P value*	Negative	Positive	*P* value
***KRAS* status, n (%)**			0.753			0.240
Wild-type	112 (92.6%)	9 (7.4%)		62 (91.2%)	6 (8.8%)	
Mutant	57 (95%)	3 (5%)		44 (97.8%)	1 (2.2%)	
***BRAF* status, n (%)**			0.110			0.334
Wild-type	146 (94.8%)	8 (5.2%)		89 (94.7%)	5 (5.3%)	
Mutant	26 (86.7%)	4 (13.3%)		17 (89.5%)	2 (10.5%)	
**MSI status, n (%)**			0.158			0.040
MSS	154 (94.5%)	9 (5.5%)		95 (96%)	4 (4%)	
MSI	19 (86.4%)	3 (13.6%)		11 (78.6%)	3 (21.4%)	

Fisher´s exact test was used for comparisons of categorical variables. Missing cases were present in faeces analyses for the following variables: *KRAS* status, 66 cases; *BRAF* status, 63 cases; MSI status, 62 cases.

**Table 4:** Associations between *P. gingivalis* and other CRC-associated bacteria in faeces and tumour tissue of CRC patients.

	Faeces			Tumour tissue		
	*P. gingivalis*			*P. gingivalis*		
	Negative	Positive	*P* value	Negative	Positive	*P* value
***F. nucleatum,* n (%)**			0.151			0.097
Low	125 (96.9)	4 (3.1)		68 (97.1)	2 (2.9)	
High	105 (92.1)	9 (7.9)		35 (87.5)	5 (12.5)	
***P. micra,* n (%)**			0.251			0.005
Low	133 (96.4)	5 (3.6)		84 (97.7)	2 (2.3)	
High	99 (92.5)	8 (7.5)		19 (79.2)	5 (20.8)	

Fischer´s exact tests were used for comparisons of categorical variables. Missing cases were present in faeces analyses for the following variables: *F. nucleatum,* 4 cases; *P. micra*, 2 cases. Missing cases in tumour tissue analyses: *F. nucleatum*, 3 cases; *P. micra*, 3 cases.

## Abbreviations

CRC: Colorectal cancer
*APC*: Adenomatous polyposis coli
*KRAS*: Kirsten rat sarcoma virus
*TP53*: Tumour protein 53
SSL: Sessile serrated lesion
*BRAF*: B-Raf
MLH1: MutL Homolog 1
MSI: Microsatellite instability
MSS: Microsatellite stability
U-CAN: Uppsala-Umeå Comprehensive Cancer Consortium
FECSU: Fecal and Endoscopic Colorectal Study in Umeå
*PGT*: Prostaglandin transporter
